# Application of an organoid-based model to explore *Helicobacter pylori–*human gastric epithelium interaction *in vitro*

**DOI:** 10.3389/fcimb.2025.1572244

**Published:** 2025-05-15

**Authors:** Byeong Min Yu, So Dam Lee, Bo Ram Hwang, Ji Seon Kim, Sungsook Yu, Ki Taek Nam, Yong Chan Lee

**Affiliations:** ^1^ Department of Internal Medicine, Institute of Gastroenterology, Yonsei University College of Medicine, Seoul, Republic of Korea; ^2^ Severance Biomedical Science Institute, Brain Korea 21 PLUS Project for Medical Science, Yonsei University College of Medicine, Seoul, Republic of Korea

**Keywords:** stomach, Helicobacter pylori, organoid, epithelial interaction, 2D model, apicalout, r-3D organoid

## Abstract

*Helicobacter pylori* infection causes histopathologic changes in gastric epithelial cells, resulting in conditions such as gastritis, gastric ulcers, and ultimately, gastric cancer. To date, various experimental models, including cell lines and animal studies, have been employed to investigate these pathological processes. However, each model presents its limitations. This study compared the re-cultured three-dimensional organoids from infected single cells, apical-out, and two-dimensional (2D) organoids models to better understand the mechanisms underlying the epithelial changes caused by *H. pylori* infection in the human stomach. Thus, we analyzed the epithelial cell responses, inflammatory mediator expressions, apical-junctional complex alterations, and *H. pylori* infection interactions in these organoid models. Moreover, we revealed that the high accessibility and experimental efficiency of the apical-out and 2D models enable easier manipulation and faster analysis of *H. pylori* infection compared to the single-cell infection model. These results indicate the potential of various organoid models in *H. pylori* infection studies to provide essential data that help in understanding the mechanisms of epithelial changes and in developing new therapeutic strategies for gastric pathology.

## 1 Introduction

Gastric cancer is one of the leading causes of cancer-related deaths globally, with a particularly high incidence in Asia (Morgan et al., 2022; Xu et al., 2020). A complex interplay of environmental, genetic, and chronic infection factors, among which *Helicobacter pylori* infection is the most crucial risk factor, affected the development of gastric cancer. The World Health Organization has classified *H. pylori* as a group 1 carcinogen because this bacterium causes chronic inflammation in the gastric mucosa, and persistent infection results in gastritis, gastric ulcers, intestinal metaplasia, and ultimately, gastric cancer (Ahn and Lee, 2015; Zhang et al., 2025). Thus, understanding the mechanism behind these pathological changes is crucial to gastric cancer prevention and treatment.

*H. pylori* infection induces structural changes and functional damage in epithelial cells through a complex host–pathogen interaction (Duan et al., 2025; Lee et al., 2014b). Specifically, damaged apical-junctional complexes and increased inflammatory mediator secretion are crucial pathological changes caused by *H. pylori* infection and are closely associated with gastric cancer development (Ansari and Yamaoka, 2019; Fazeli et al., 2016). In particular, the CagA protein, a crucial virulence factor of *H. pylori*, plays a crucial role in disrupting cell signaling and promoting oncogenic transformation, further contributing to gastric cancer progression. Therefore, an appropriate model is required to investigate the early stages of *H. pylori* infection and its effects on epithelial cells and tissues. Traditional animal models and cell culture techniques contribute to *H. pylori* research; however, these models do not perfectly replicate the epithelial structure and environment of the human body. To address these limitations, three-dimensional (3D) organoids, which more accurately replicate the structure and function of organs and are suitable for studying cell-to-cell interactions, have been recently developed (Huch et al., 2013; Pleguezuelos-Manzano et al., 2020). Derived from adult stem cells or induced pluripotent stem cells (iPSCs), 3D organoids replicate the physiological and pathological changes of gastric epithelial cells, making them a crucial tool in gastric cancer and *H. pylori* infection research. However, despite these advantages, they are limited concerning access to their apical surface. Apical-out and 2D organoid models have been developed to overcome these drawbacks.

The apical-out model is established to expose the apical surface of epithelial cells to the outside, enabling direct assessments of interactions with pathogens. Unlike traditional 3D organoids, investigating mechanisms related to epithelial polarity is particularly suitable. This model facilitates the precise analysis of the initial infection process with pathogens such as *H. pylori* (Co et al., 2021). Consisting of a single-cell layer, 2D organoids provide a simpler structure suitable for studying basic cell functions and interactions. These models are useful for investigating cellular interactions, differentiation, and cell migration while effectively recapitulating the fundamental characteristics of epithelial cells. Further, 2D organoids can be maintained and differentiated in a more physiologically relevant environment when combined with air-liquid interface (ALI) culture systems. The ALI system enables epithelial cells to be exposed to air without direct contact with the underlying matrix, which mimics more natural conditions. This setup improves the functionality of the organoids, enabling them to better replicate epithelial barrier functions and host–pathogen interactions (Boccellato et al., 2019; Uotani et al., 2019).

In this study, we utilized the re-cultured 3D organoids from infected single cells (r-3D), apical-out, and 2D organoid models to investigate the effects of *H. pylori* infection on gastric epithelial cells. We aimed to investigate the advantages and limitations of each model and propose the optimal model for studying *H. pylori* infection, thereby providing novel information about the mechanisms underlying gastric cancer development.

## 2 Materials and methods

### 2.1 Ethical statement

This study used human tissue specimens obtained from patients with gastric cancer at Severance Hospital. The institutional review board (IRB) of Severance Hospital approved this study (IRB numbers: 4-2018-0676). All participants provided informed consent before tissue sample collection.

### 2.2 3D organoid culture

Gastric tissue samples were collected from patients diagnosed and treated for gastric cancer at Severance Hospital, Yonsei University, from 2018 to 2022. Gastric biopsy samples collected via endoscopy were processed as follows. The biopsied tissues were washed three times with Dulbecco’s phosphate-buffered saline (PBS), incubated in a dissociation solution for 15 min at room temperature (RT), and dissociated into smaller fragments using a 1-mL pipette tip. Tissues were then washed three more times with Dulbecco’s PBS, and the remaining pellet was filtered to collect small gastric glands. To form organoids, the small gastric cells were mixed with Matrigel at a 20 μL per 10 glands ratio. The mixture was then dispensed into 48-well plates and solidified in a 37°C incubator for 10 min. After solidification, the culture medium was added and replaced every 3 days. The culture medium consisted of advanced DMEM/F12 supplemented with penicillin, streptomycin, 10 mmol/L of HEPES (Invitrogen, MA, USA), glutamax (Invitrogen), 1 × B27 (Invitrogen), 1 mM of N-acetylcysteine (Sigma-Aldrich, MO, USA), 50 ng/mL of epidermal growth factor (EGF, Invitrogen), 10% Noggin-conditioned medium, 10% R-spondin1-conditioned medium, 50% Wnt-conditioned medium, 200 ng/mL of FGF10 (PeproTech, NJ, USA), 1 nM of gastrin (Tocris, UK), 2 μM of TGFβi (A-83-01, Sigma-Aldrich), and optionally 10 mM of nicotinamide (Sigma-Aldrich). Matrigel domes were treated with 10 μM of RHOKi (Y-27632, Sigma-Aldrich). The protocol for the gastric organoid culture was developed based on published research (Huch et al., 2013; Pleguezuelos-Manzano et al., 2020; Vlachogiannis et al., 2018).

### 2.3 3D organoids re-cultured from 3D-derived single cells after infection (r-3D)

A Pasteur pipette in PBS was used to mechanically dissociate 1–2-week-old organoids removed from Matrigel to expose the apical membrane. Organoids were then resuspended in a coculture antibiotic-free medium. The cell suspension was centrifuged and resuspended in TrypLE (Invitrogen), followed by a 5-minute incubation at 37°C. After complete dissociation, the cells were counted utilizing a microscope and hemocytometer to calculate the cell density (cells/mL).

*H. pylori* was harvested in PBS and stained with a fluorescent dye (Cell Trace Far Red, 1 μM, Invitrogen) at a 1:1000 ratio. A bacterial culture with an OD600 value of 0.3–0.9 was then added to the cells at a multiplicity of infection (MOI) of 1:100 for 4 h. Subsequently, the organoids were harvested, washed to remove unbound bacteria, and resuspended in Matrigel. Matrigel-embedded organoids were then bathed in an organoid growth medium.

Matrigel contained 50 μg/mL of gentamicin to suppress bacterial growth outside the organoid lumen. Gentamicin inhibited the survival of Gram-negative bacteria, including *H. pylori*, under these conditions in the external environment. However, bacteria located in the lumen of the organoids survived related to the protection provided by the Matrigel and tissue structure.

*H. pylori*-infected organoids were further cultured for 24–48 h. Bacteria in the external environment (with gentamicin) could not survive, whereas those located in the apical region within the organoid lumen remained viable under these conditions.

### 2.4 Generation of the apical-out organoid model

The apical-out organoid induction method was based on a published study (Co et al., 2021). Organoids cultured for >2 week were removed from Matrigel domes and incubated in a solution containing 5 mM of ethylenediaminetetraacetic acid (EDTA) (E9884, Sigma, Japan) in PBS without Ca^2+^ or Mg^2+^ at 4°C on a rotary platform for 1 h for the apical-out organoid models used in *H. pylori* infection. The organoid suspension was centrifuged at 200 g for 3 min at 4°C and the supernatant discarded before resuspending the pellet in the same medium used for the 3D organoid culture and incubated at 37°C with 5% CO_2_ for 48 h. Finally, apical-out organoids were infected with fluorescently labeled *H. pylori* for 8 h at an MOI of 100, enabling the bacteria to access the luminal surface of the exposed epithelium.

### 2.5 Transition to 2D organoids

For the transition to a 2D format, approximately 2–2.5 × 10⁵ cells derived from 3D organoids cultured for >1 week were seeded onto collagen-coated transwell inserts (15 µg/cm²) with 200 µL of medium in 24-well plates for the transition to a 2D format. The space between the filter and the well was filled with 400 µL of medium. This setup was maintained in a humidified incubator at 37°C with 5% CO_2_. On day 3, the medium above the cells on the filter was removed and that below the filter was replaced by 500 µL twice weekly to develop an ALI culture. The 2D organoid (mucosal) culture medium was similar to the 3D organoid medium and included penicillin, streptomycin, 10 mmol/L of HEPES (Invitrogen), glutamax (Invitrogen), 1 × B27 (Invitrogen), 1 × N2 (gibco), 20 ng/mL of EGF (Invitrogen), 150 ng/mL of noggin (PeproTech), 25% R-spondin1-conditioned medium, 50% Wnt-conditioned medium, 150 ng/mL of FGF10 (PeproTech), 10 nM of gastrin (Tocris), 1 μM of TGFβi (A-83-01, Sigma-Aldrich), 10 mM of nicotinamide (Sigma), and 7.5 μM of RHOKi (Y-27632, Sigma-Aldrich). RHOKi was removed after 3 days. The protocol for 2D organoid culture was based on published research. *H. pylori* infection of 2D organoids was initiated on day 14 after 2D organoid generation. The bacterial load was identified by measuring the optical density at 600 nm (MOI = 1:100 and 20) (Boccellato et al., 2019; Hoffmann et al., 2021; Roodsant et al., 2020).

### 2.6 *H. pylori* culture and infection

*H. pylori* strains 60190 (CagA+, 49503, ATCC), 11637 (CagA+, 43504, ATCC), and ΔcagA (cagA-deficient isogenic mutant of 60190, ATCC 49503) were cultured on agar plates containing 10% horse serum at 37°C under a microaerobic atmosphere utilizing the Campy Container system (BBL, USA). Bacteria were harvested in Dulbecco’s PBS (pH: 7.4) and added to host cells at MOI of 100 and 20, with optical density at 600 nm (OD600) measured for bacterial quantification for infection. PBS was added to the cells and incubated for the same period as a control. Professor M. Hatakeyama (University of Tokyo, Japan) kindly provided the *H. pylori* ΔCagA strain (Kikuchi et al., 2012; Lee et al., 2014a).

### 2.7 Enzyme-linked immunosorbent assay

The interleukin (IL)-8 concentration in the culture supernatants collected from the three organoid models was quantified using the direct-sandwich ELISA method. To ensure the reliability of the findings, each sample was analyzed in quintuplicate. IL-8 quantification was conducted using the human CXCL8/IL-8 DuoSET (DY208-05, R&D Systems, MN, USA) and the DuoSET Ancillary Reagent Kit (DY008, R&D Systems). ELISA analyses were conducted following the manufacturer’s standard protocols, and the optical absorbance was measured at 450 nm utilizing a VersaMax microplate reader.

### 2.8 RNA extraction and quantitative reverse transcription polymerase chain reaction

QIAzol lysis reagent (QIAGEN 79306) was used to extract RNA from 2D human gastric organoids, following the manufacturer’s instructions. Maxime RT PreMix (25081, Intronbio, Korea) was utilized to synthesize cDNA from 1 μg of RNA. Quantitative PCR amplification was performed using SYBR Green (CMQSR1000, Cosmogenetech, Korea) following the manufacturer’s protocol. The following specific primers used in this study (listed below) were synthesized by Bioneer:

- GAPDH (F): CGACCACTTTGTCAAGCTCA (20 mer)
- GAPDH (R): AGGGGTCTACATGGCAACTG (20 mer)
- LGR5 (F): CTCCCAGGTCTGGTGTGTTG (20 mer)
- LGR5 (R): GCTCGCAATGACAGTGTGTG (20 mer)
- MUC5AC (F): GGAGGTGCCCACTTCTCAAC (20 mer)
- MUC5AC (R): CTTCAGGCAGGTCTCGCTG (19 mer)
- MUC6 (F): CAGCTCAACAAGGTGTGTGC (20 mer)
- MUC6 (R): TGGGGAAAGGTCTCCTCGTA (20 mer)
- Ki67 (F): CACACTGTGTCGTCGTTTGT (20mer)
- Ki67 (R): ATTCCCAAGAGACCAAGGCA (20mer)

### 2.9 Transepithelial electrical resistance

To assess changes in the electrical resistance based on 2D organoid cell density, we measured the TEER of the epithelial cell layers. TEER was evaluated with an EVOM2 (WPI, UK) device, which measured the electrical resistance of the epithelial cell layer and the culture medium. The measured resistance includes both the epithelial layer and the medium; thus, blank resistance measurements were subtracted to accurately identify the electrical resistance of the organoid epithelium. This enabled the precise measurement of TEER levels corresponding to changes in cell density and played a crucial role in evaluating the barrier function of the organoid epithelium.

### 2.10 Scanning electron microscopy

For SEM, 2D organoid samples were fixed in Karnovsky’s fixative (2% glutaraldehyde, 2% paraformaldehyde (PFA), and 0.1 M of phosphate buffer) for 24 h. The samples were then washed twice with 0.1 M of phosphate buffer, postfixed with 1% OsO₄, and dehydrated with a graded ethanol series. Subsequently, the samples were processed employing a critical point dryer (LEICA EM CPD300), coated with platinum using an ion sputter coater (LEICA EM ACE600), and imaged utilizing a MERLIN scanning electron microscope (ZEISS).

### 2.11 Immunohistochemistry and immunofluorescence

Formalin-fixed paraffin-embedded slides were sequentially deparaffinized and rehydrated in 100%, 90%, and 70% ethanol for immunostaining. The antigen was retrieved by incubating the slides in an antigen retrieval solution (S169984-2, Dako, Denmark) with a pressure cooker. The slides were blocked at RT in a humidified chamber for 1–2 h using a cell-free protein blocker (X0909, Dako) after two PBS washes. Additional treatment with the mouse-on-mouse reagent (Vector) was performed before the primary antibody incubation for mouse tissue samples. The slides were incubated overnight at 4°C with primary antibodies. Secondary antibodies conjugated with Texas Red (Invitrogen), fluorescein isothiocyanate (FITC, Invitrogen), Cy3 (Invitrogen), or Cy5 (Invitrogen) were used to detect the respective primary antibodies for immunofluorescence staining. Nuclear staining was performed using 6-diamino-2-phenylindole (DAPI, Sigma, Japan). A confocal (LSM 770, ZEISS, Germany) and an immunofluorescence microscope (EVOS-M5000, Thermo Fisher Scientific) were used for imaging.

### 2.12 Transferase dUTP nick end labeling Assay

To evaluate apoptosis, a terminal deoxynucleotidyl TUNEL assay was conducted using the TUNEL Assay Kit—BrdU-Red (ab66110, Abcam) following the manufacturer’s instructions. Further, 2D organoids were fixed in 4% PFA at RT for 30 min and then embedded in paraffin blocks. Subsequently, 5-μm thick sections were prepared on slides.

The slides were dried at 60°C for 1 h, deparaffinized using xylene, and rehydrated through a graded ethanol series (100%, 95%, and 70%). After rehydration, the sections were permeabilized with 0.1% triton X-100 in PBS for 10 min and washed with PBS. The sections were then incubated with a labeling reaction mixture containing terminal deoxynucleotidyl transferase and BrdU at 37°C in a humidified chamber for 60 min. After incorporating BrdU into fragmented DNA, the sections were incubated with an anti-BrdU antibody conjugated with a red fluorophore for 30 min at RT in the dark. Finally, the sections were washed with PBS, counterstained with DAPI (4’,6-diamidino-2-phenylindole) for 10 min, and mounted with an antifade medium.

Images were acquired utilizing an immunofluorescence microscope (EVOS-M5000, Thermo Fisher Scientific) and analyzed with ImageJ software. The percentage of TUNEL-positive cells was quantified by counting BrdU-positive nuclei associated with the total DAPI-stained nuclei in multiple randomly selected fields.

### 2.13 Antibodies

The following primary antibodies were commercially purchased and utilized for immunofluorescence analyses: ZO-1 (339100, Invitrogen), *H. pylori* (NBP2-29479, Novus), Pepsinogen C (NBP1-91011, Novus), Muc5ac (ab77576, Abcam), MUC6 (ab212646, Abcam), E-cadherin (ab40772, Abcam), Snail (3895S, CST), Ki67 (sc-23900, Santa Cruz), Muc2 (MA5-12345, Invitrogen), and CagA (sc-28368, Santa Cruz).

### 2.14 Statistical analysis

GraphPad Prism version 10.3.1 (GraphPad Software Inc., La Jolla, CA) was used for statistical analyses. The two groups were compared using the independent Student’s *t*-test. One- or two-way analysis of variance (ANOVA) was conducted for comparisons among multiple groups, followed by Tukey’s or Sidak’s multiple comparison tests to identify statistical significance. All data are expressed as the mean ± standard error of the mean (SEM), and a *p*-value of <0.05 indicates statistical significance.

## 3 Results

### 3.1 *In vitro* growth and differentiation of patient-derived gastric organoids

**Figure 1:**
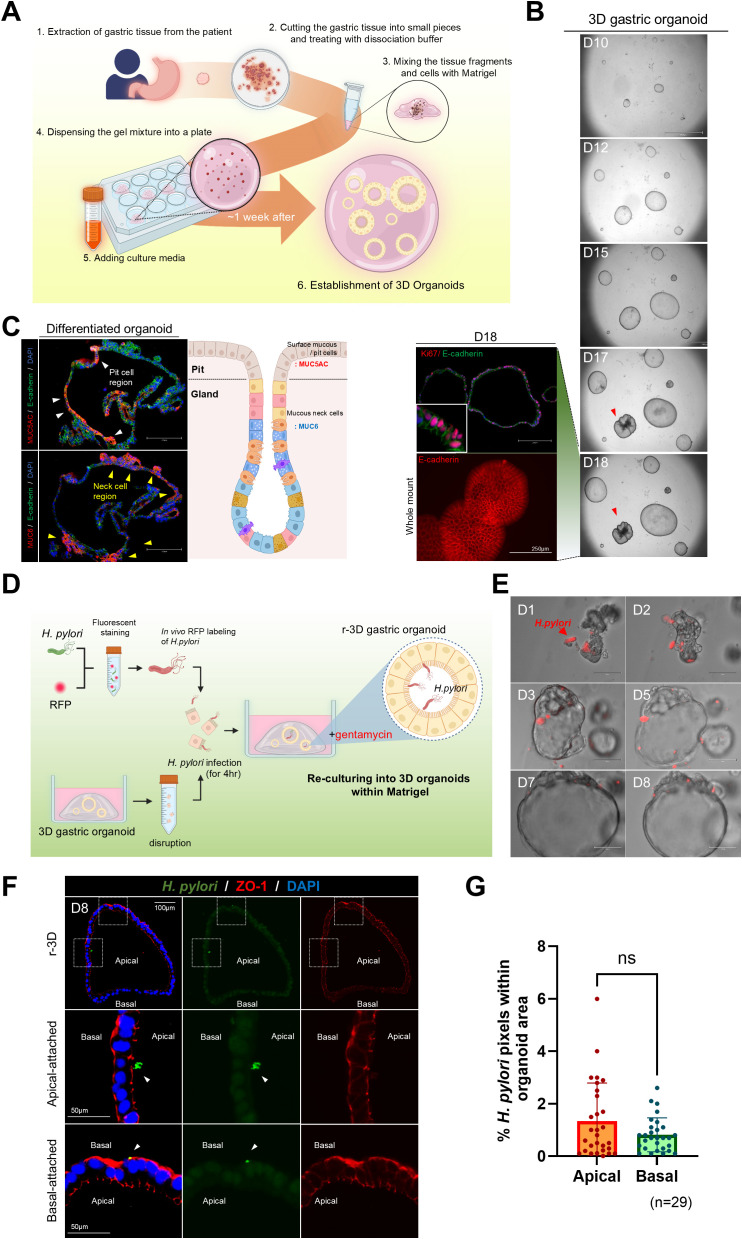
Re-culturing of *Helicobacter pylori*-infected single cells derived from three-dimensional organoids. **(A)** Schematic representation of the method for generating gastric organoids from patient-derived samples. **(B)** *In vitro* growth of patient-derived gastric organoids. The scale bar indicates 2100 µm. Whole-mount staining and Ki67 staining of day 18 organoids. The scale bar indicates 250 µm. **(C)** Expression of gastric-specific markers, MUC5AC (red) and MUC6 (red), in patient-derived gastric organoids. Scale bar = 250 µm. **(D)** Schematic of *H. pylori* infection within the lumen of mechanically disrupted 3D organoids. **(E)** Bright-field images illustrating coculture of fluorescently labeled *H. pylori* (red) and r-3D gastric organoids. **(F)** Immunofluorescence images of *H pylori* attachment sites in day 8 r-3D gastric organoids. *H. pylori* (green), ZO-1 (red), DAPI (blue). Scale bars = 100 µm and 50 µm. **(G)** Graph illustrating the percentage of pixels occupied by *H. pylori* in the apical and basal organoid regions. Statistical analysis was conducted using a two-tailed unpaired Student’s *t*-test: ns > 0.05. (n=29).

Gastric glands isolated from the body region of patient-derived stomach tissue were embedded in Matrigel and cultured in a complete organoid growth medium. These glands grew into spherical organoids (**Figure 1A**). Budding structures began to be observed by day 18, and Ki67 immunofluorescence staining confirmed active cell proliferation (**Figure 1B**). To visualize cellular differentiation, we conducted immunofluorescence staining. After 18 days, the organoids were further cultured in a differentiation medium without Wnt3a and R-spondin1 for 2 days. This revealed MUC5AC (surface mucous cell marker) and MUC6 (neck cell marker) expressions in the organoids (**Figure 1C**). These results demonstrated that the gastric organoids exhibit well-characterized gastric pit and gland structures representative of gastric lineage features.

### 3.2 Assessment of *H. pylori* attachment to apical and basal surfaces in r-3D

To promote *H. pylori* attachment to the apical surface of 3D gastric organoids, the organoids were mechanically disrupted and exposed to a red fluorescent protein (RFP)-labeled *H. pylori* for 4 h. Organoids were then re-embedded in Matrigel and re-cultured with gentamicin (50 μg/mL) in the medium to remove externally attached *H. pylori* (**Figure 1D**). Upon re-culture, *H. pylori*-infected gastric organoids regained a spheroid shape by day 3, and *H. pylori* remained observable within the luminal space for up to 8 days (**Figure 1E**). Immunofluorescence staining confirmed *H. pylori* attachment to the organoid, and the analysis of the pixel percentage occupied by *H. pylori* within the organoid region quantified the infection level (**Figure 1F**). However, staining for the apical marker ZO-1 demonstrated no significant difference in *H. pylori* attachment between the apical and basal surfaces of the organoids (n = 20) (**Figure 1G**). These results emphasize the limitations of existing methods for causing *H. pylori* attachment to the apical surface and highlight the need for improved infection models.

### 3.3 Polarity reversal and expression of apical epithelial markers in gastric organoids

**Figure 2:**
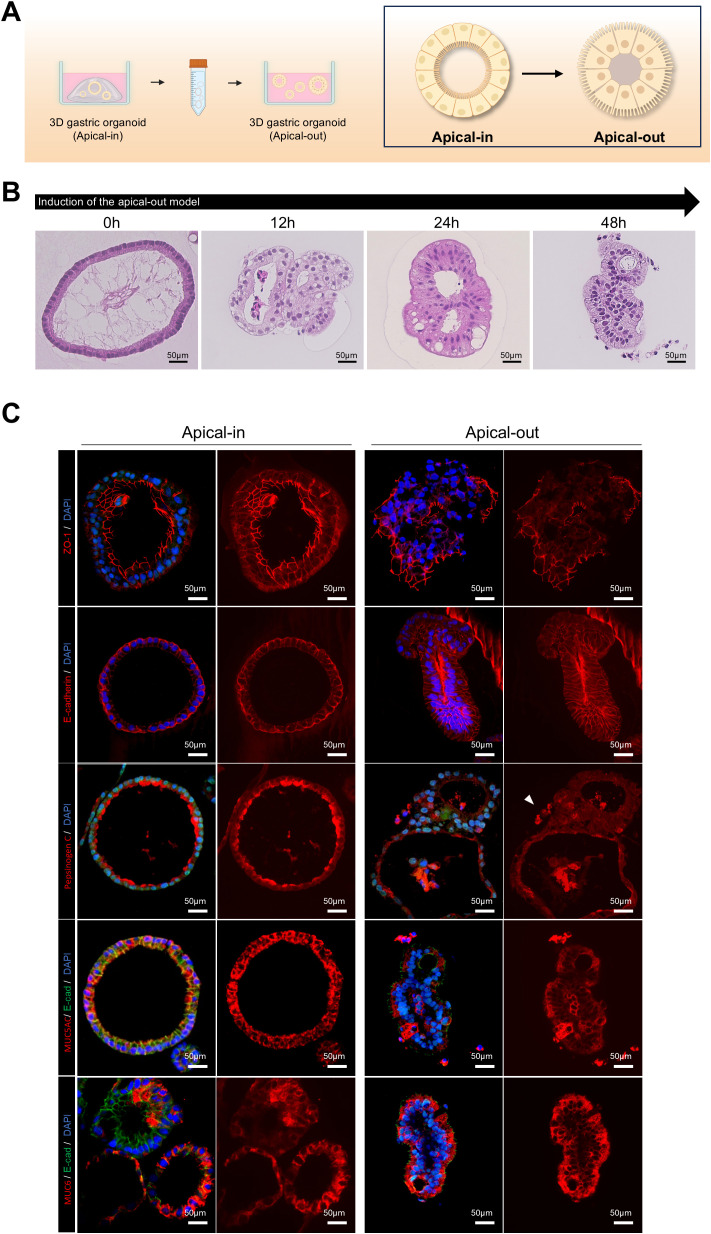
Induction of polarity reversal and apical epithelial marker expression in 3D gastric organoids. **(A)** Schematic depicting the polarity reversal process from apical-in to apical-out configuration in patient-derived 3D gastric organoids. **(B)** Hematoxylin and eosin (H&E) staining images of apical-out organoids at different time points after induction. The scale bar indicates 50 µm. **(C)** Fluorescence staining images of apical-in and apical-out organoids demonstrating ZO-1 (apical marker and tight junction protein), E-cadherin (adherens junction protein), pepsinogen (chief cell marker), MUC5AC (surface mucous cell marker), and MUC6 (neck cell marker). The scale bar indicates 50 µm. Organoids were cultured for over 2 weeks before analysis.

Previous studies (Co et al., 2021) revealed a method for polarity reversal in intestinal organoids, exposing the apical surface externally, thereby providing a valuable model for pathogen coculture systems. To induce polarity reversal and expose the apical surface, gastric organoids were removed from Matrigel and incubated in 5 mM of EDTA solution for 1 h. They were then suspended and cultured in a growth medium for 48 h to induce polarity reversal (**Figure 2A**). After polarity reversal, the nuclei migrated toward the lumen (**Figure 2B**), and ZO-1, which is an apical marker, localized to the external surface of the organoids, as confirmed by 3D immunofluorescence imaging. Further, E-cadherin staining revealed cytoskeletal reorganization. We observed pepsinogen C, MUC5AC, and MUC6 secretion from the organoid interior to the exterior (**Figure 2C**). These results suggest that the apical-out gastric organoid model provides a suitable environment for studying pathogen-host interactions.

### 3.4 *H. pylori infection* and epithelial interactions in apical-out gastric organoids

**Figure 3:**
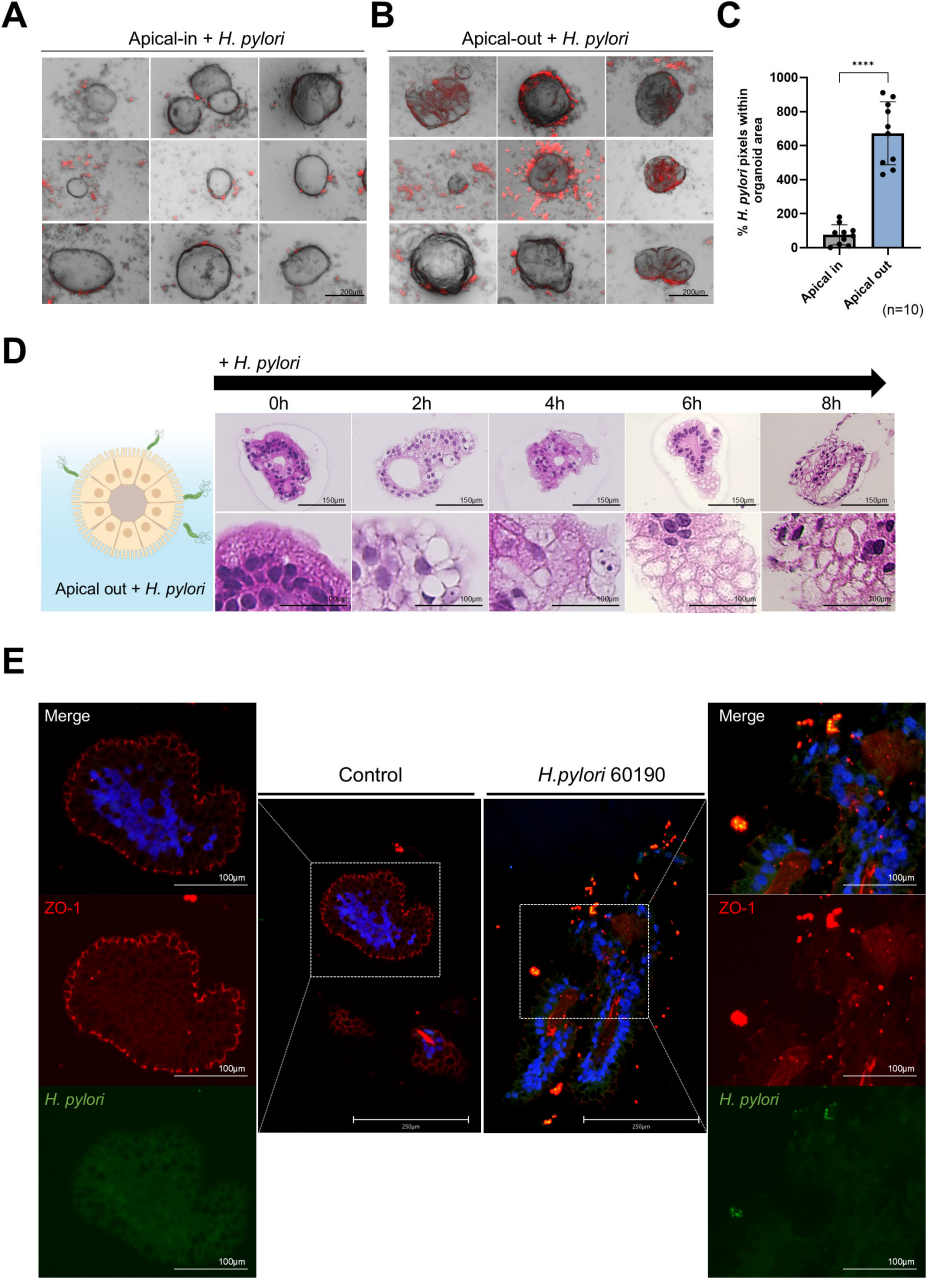
*H pylori* infection and epithelial cell interaction in apical-out organoids. **(A)** Bright-field and fluorescence images after infecting apical-in organoids with *H. pylori* 60190 for 8 h *H. pylori* (red), organoid (B.F.). The scale bar indicates 200 µm. **(B)** Bright-field and fluorescence images after infecting apical-out organoids with *H pylori* 60190 for 8 h. The scale bar indicates 200 µm. **(C)** Graph illustrating the percentage of pixels occupied by fluorescently labeled *H. pylori* 60190 in apical-in and apical-out organoids. Statistical analysis was conducted using a two-tailed unpaired Student’s *t*-test: ****P < 0.0001 (n = 10). **(D)** H&E staining images of apical-out organoids infected *with H. pylori* 60190 for 0, 2, 4, 6, and 8 h and after 48 h of induction. The scale bar indicates 150 µm. **(E)** Immunofluorescence images illustrating changes in the apical protein ZO-1 after infecting apical-out organoids with *H. pylori* 60190 for 8 h Scale bars = 250 µm and 100 µm.

This study compared the attachment efficiency of *H. pylori* and epithelial changes after infection in organoids with reversed polarity. Apical-in and apical-out organoids were prepared and infected with RFP-labeled *H. pylori* at the same concentration for 8 h. After washing with dPBS to remove unbound *H. pylori*, the RFP fluorescence intensity in organoids infected with *H. pylori* was measured. Apical-out organoids (**Figure 3B**) exhibited significantly higher *H. pylori* attachment than Apical-In organoids (**Figures 3A, C**). We then analyzed the morphological changes in apical-out organoids after *H. pylori* infection using hematoxylin and eosin (H&E) staining at 0, 2, 4, 6, and 8 h postinfection. Goblet cell-like structures markedly increased after 2 h of infection; changes which became more pronounced over time (**Figure 3D**). To assess the effect of *H. pylori* infection on the apical-junctional structure of the organoids, ZO-1 protein expression was analyzed with immunofluorescence staining. Lower ZO-1 expression and disruption of the apical-junctional structure were observed at 8 h postinfection (**Figure 3E**). These results indicate that *H. pylori* infection in apical-out organoids compromises the epithelial polarity and apical-junctional complex stability.

### 3.5 Cellular structural changes and decreased electrical resistance in a 2D gastric organoid model after *H. pylori infection*

**Figure 4:**
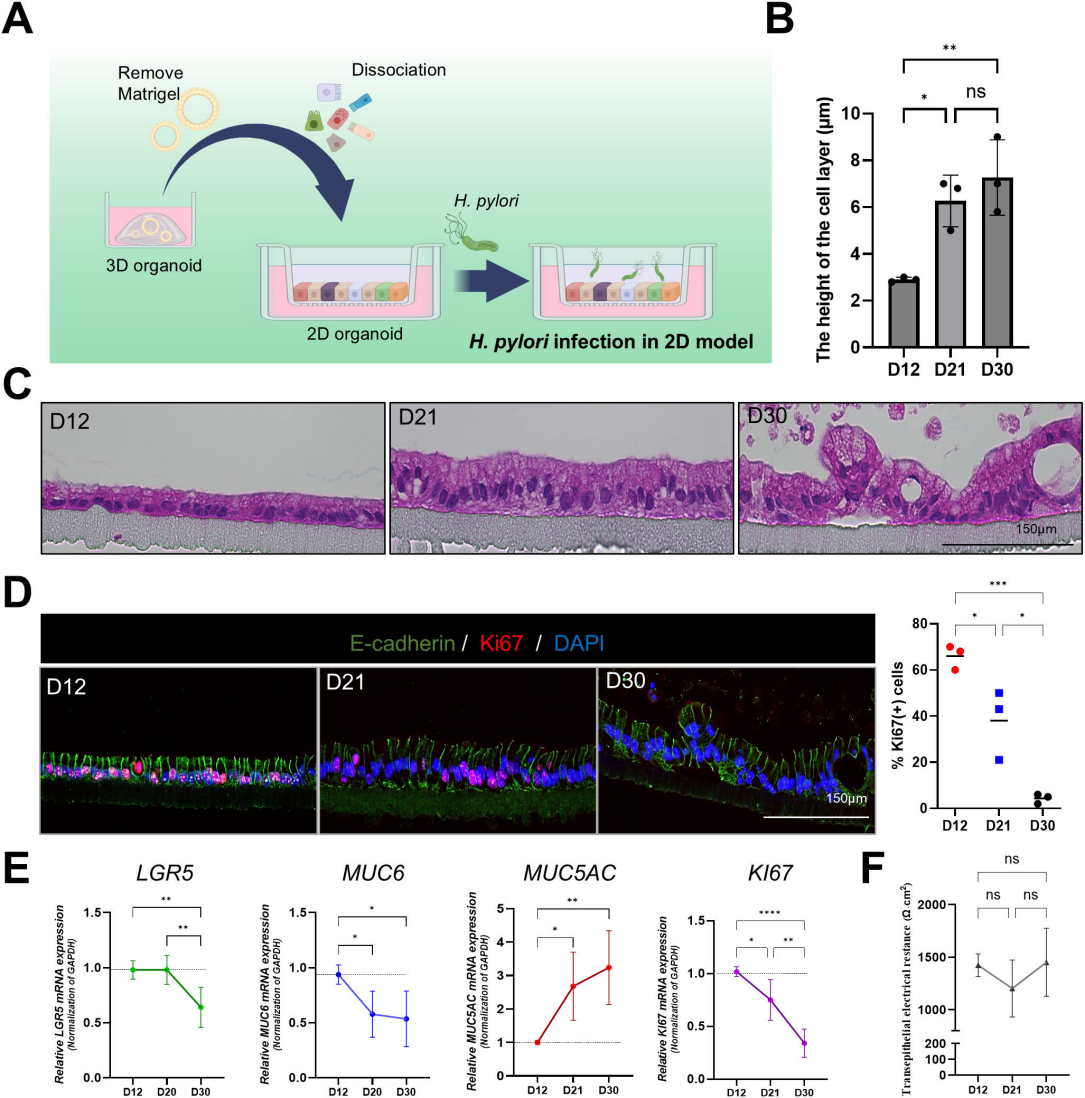
Structural and functional characterization of 2D gastric organoids on filters. **(A)** Schematic representation of the process of transforming patient-derived 3D gastric organoids into 2D organoids. **(B)** Changes in the cell monolayer thickness in 2D organoids. Statistical analysis was conducted using one-way ANOVA with Tukey’s multiple comparisons: **P < 0.05; **P < 0.01 (n = 3). **(C)** H&E staining of 2D organoid monolayers on culture days 12, 21, and 30. The scale bar indicates 150 µm. **(D)** Fluorescence staining of Ki67-positive proliferating cells in 2D organoids on culture days 12, 21, and 30. The scale bar indicates 150 µm. Right: expression levels of Ki67 at each time point. Statistical analysis was conducted using one-way ANOVA with Tukey’s multiple comparisons: *P < 0.05; **P < 0.01; ***P < 0.001 (n = 3). **(E)** mRNA expression levels of Lgr5, MUC6, MUC5AC, and Ki67 in 2D organoids at different time points. Statistical analysis was conducted using one-way ANOVA with Tukey’s multiple comparisons: *P < 0.05; **P < 0.01; ***P < 0.001 (n = 5). **(F)** Changes in transepithelial electrical resistance (TEER) of 2D organoids according to culture time. Statistical analysis was conducted using one-way ANOVA with Tukey’s multiple comparisons: ns >0.05 (n = 3).

**Figure 5:**
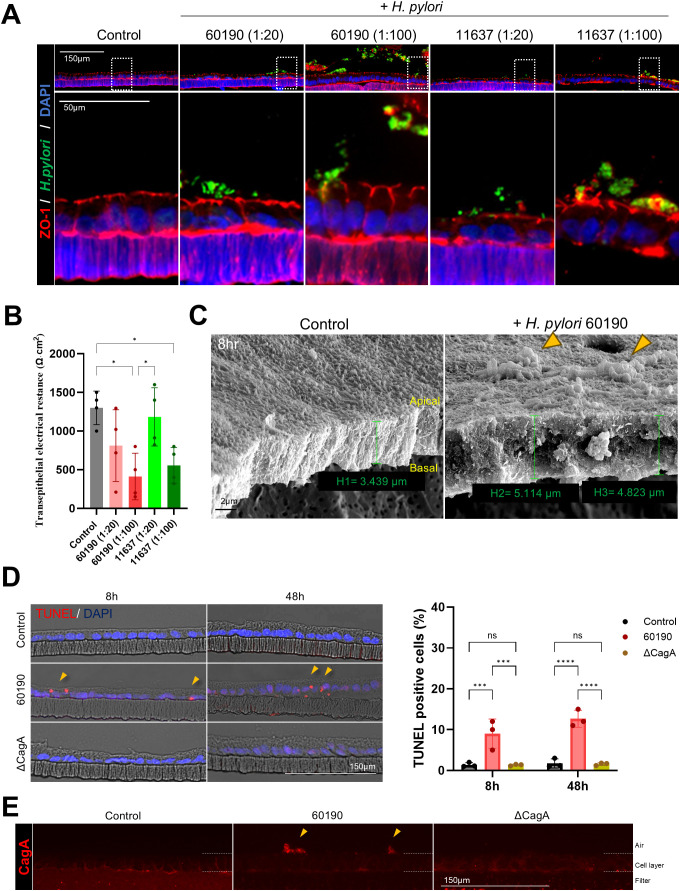
Structural changes and cellular responses of 2D gastric organoids on filters after *H. pylori* infection. **(A)** Immunofluorescence images demonstrating changes in the apical protein ZO-1 after infecting 2D organoids with *H. pylori* strains 60190 and 11637 at MOI of 1:20 and 1:100 for 8 h Scale bars = 150 µm (top) and 50 µm (bottom). **(B)** TEER measurements of 2D organoids before and after *H. pylori* infection. Statistical analysis was conducted using one-way ANOVA and Tukey’s multiple comparisons test: *P < 0.05 (n = 4). **(C)** Scanning electron microscopy (SEM) images of 2D organoids infected with *H pylori* for 8 h The scale bar indicates 2 µm. **(D)** TUNEL assay analysis to evaluate cell apoptosis in 2D organoids infected with CagA-positive strain (60190) and CagA-negative strain (ΔCagA) for 8 h and 48 h The scale bar indicates 150 µm. The bar graph on the right illustrates the quantification of TUNEL-positive cells. Statistical analysis was conducted using two-way ANOVA and Tukey’s multiple comparisons test: ***P < 0.001, ****P < 0.0001 (n = 3). **(E)** Immunofluorescence staining for CagA expression in 2D organoids infected with CagA-positive strain (60190) and CagA-negative strain (ΔCagA). The scale bar indicates 150 µm.

In addition to the apical-out model, we used a 2D gastric organoid (mucosoid) model that mimics the key characteristics of the gastric mucosa. This model creates a single-cell layer on a filter and secretes mucus from the apical surface. Employing an air-liquid interface (ALI) culture system, it closely resembles the *in vivo* gastric environment (**Figure 4A**). Further, we analyzed the structural changes occurring in 2D organoids over time. Compared to day 12 (D12) of culture, the thickness of the monolayer significantly increased by day 21 (D21) (**Figures 4B, C**), whereas the proportion of Ki67-positive cells decreased (**Figure 4D**). Moreover, Lgr5, Muc6, and Ki67 mRNA expression significantly decreased at D30 compared with D12, whereas MUC5AC expression increased (**Figure 4E**). These results indicate a natural differentiation over prolonged culture even in the absence of differentiation medium. TEER measurements indicated stable cell–cell adhesion, with resistance values of >1000 Ω·cm² at all-time points (**Figure 4F**). For the *H. pylori* infection experiment, 2D gastric organoids cultured for 2 weeks were infected with *H. pylori* strains 60190 and 11637 at MOI of 1:20 and 1:100 for 8 h. Immunofluorescence staining revealed co-localization of *H. pylori* with ZO-1 on the apical surface of the organoids (**Figure 5A**), indicating an interaction between *H. pylori* and the apical-junctional structures. After infection, TEER measurements revealed a significant decrease in electrical resistance in 2D organoids infected with *H. pylori* at MOI of 1:100, indicating weakened cell–cell adhesion caused by *H. pylori* infection (**Figure 5B**). Further, SEM analysis revealed changes in the monolayer thickness and localization of *H. pylori* in the infected group (**Figure 5C**). To evaluate cell apoptosis in 2D organoids infected with *H. pylori*, a TUNEL assay was conducted, emphasizing the role of CagA. A significant increase in TUNEL-positive cells was observed in 2D organoids infected with the CagA-positive strain 60190 at both 8 h and 48 h. In contrast, 2D organoids infected with the CagA-negative strain ΔCagA demonstrated no significant difference compared to the control group (**Figures 5D, E**). These results indicate that CagA plays a crucial role in improving the pathological effects of *H. pylori* infection, with CagA presence contributing to increased cell apoptosis and reflecting the pathological changes observed in the gastric mucosa.

### 3.6 Comparison of cellular polarity and inflammatory responses in r-3D, apical-out models, and 2D gastric organoid models

**Figure 6:**
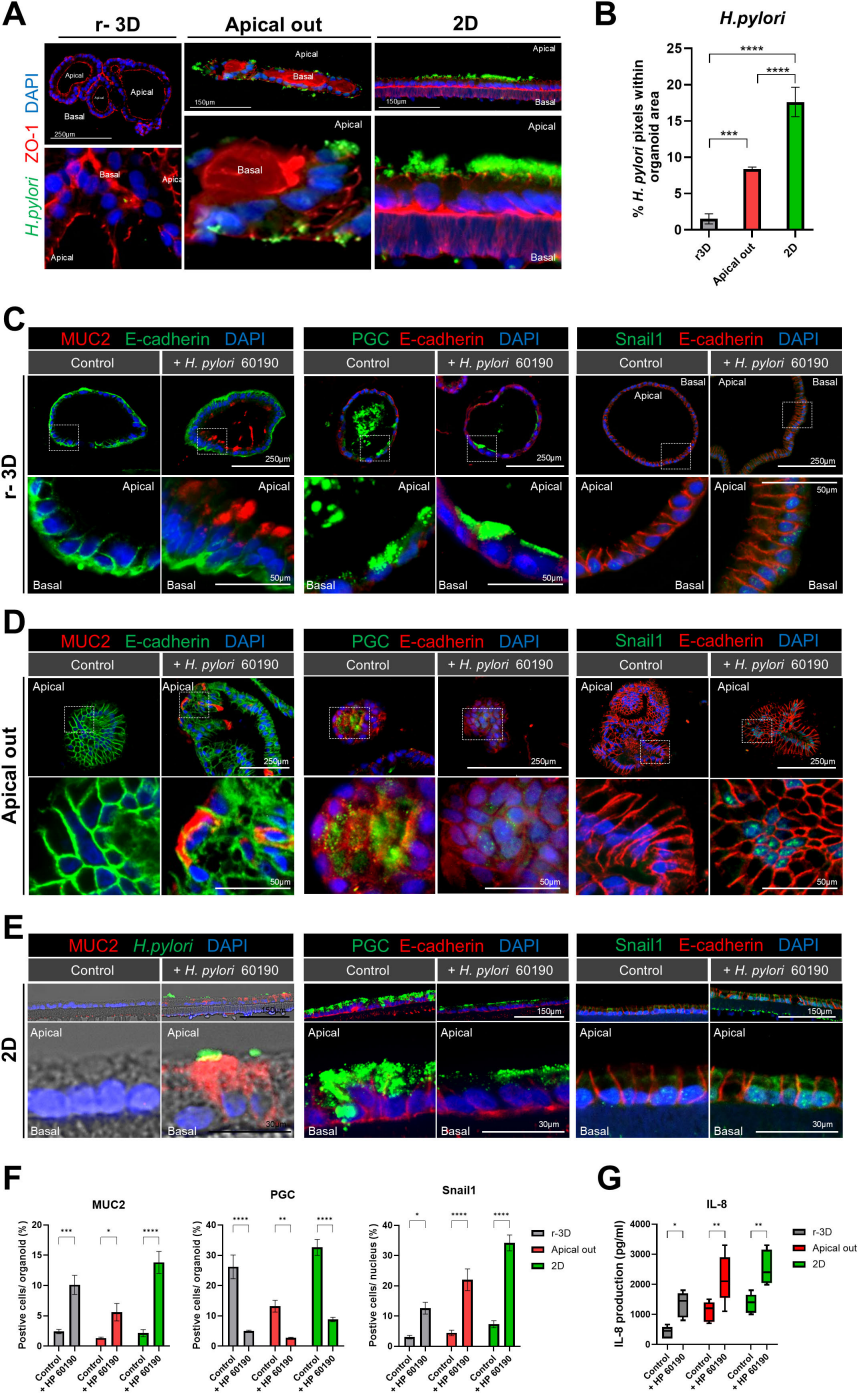
Cellular polarity changes and inflammatory response: comparison of r-3D, apical-out, and 2D models. **(A)** Immunofluorescence images illustrating *H. pylori* 60190 infection in the r-3D organoids, apical-out, and 2D models. The scale bar indicates 250 µm and 150 µm. **(B)** Graph representing the percentage of pixels occupied by *H. pylori* 60190 within r-3D organoids, apical-out, and 2D organoids. Statistical analysis was conducted using one-way ANOVA with Tukey’s multiple comparisons: *P < 0.05; **P < 0.01; ***P < 0.001 (n = 5). **(C)** Immunofluorescence images demonstrating the expression of MUC2 (intestinal metaplasia marker), pepsinogen C (PGC, chief cell marker), and Snail1 (EMT transcription factor) in r-3D organoids. Scale bars = 250 µm (top) and 50 µm (bottom). **(D)** Immunofluorescence images illustrating MUC2, pepsinogen C, and Snail1 expression in apical-out organoids. Scale bars = 250 µm (top) and 50 µm (bottom). **(E)** Immunofluorescence images showing MUC2, pepsinogen C, and Snail1 expression in the 2D organoids. Scale bars = 150 µm (top) and 30 µm (bottom). **(F)** Graph representing the percentage of positive cells in r-3D, apical-out, and 2D organoids. Statistical analysis was conducted using two-way ANOVA with Sidak’s multiple comparisons: *P < 0.05; **P < 0.01; ***P < 0.001 (n = 5). **(G)** ELISA graph illustrating IL-8 production in *H. pylori* 60190-infected organoids. Statistical analysis was conducted using two-way ANOVA with Sidak’s multiple comparisons: *P < 0.05; **P < 0.01 (n = 5).

We compared changes in cellular polarity and inflammatory responses among r-3D, apical-out, and 2D gastric organoid models after *H. pylori* infection. Hence, a significant increase in the *H. pylori* pixel percentage was observed in both apical-out and 2D organoids compared to the r-3D organoids, with the highest percentage seen in the 2D organoid model (**Figures 6A, B**). Immunofluorescence staining was conducted to evaluate the effects of *H. pylori* infection on the expression of MUC2 (intestinal metaplasia marker), pepsinogen C (PGC, chief cell marker), and snail1 (epithelial-mesenchymal transition [EMT] transcription factor) in each organoid model. The r-3D organoids demonstrated increased MUC2 secretion at the apical surface, decreased PGC expression, and elevated nuclear Snail1 expression after *H. pylori* infection (**Figure 6C**). However, due to the inherent limitations of the 3D model, excluding the influence of external *H. pylori* located outside the organoid lumen was difficult. In apical-out organoids, similar changes in MUC2, PGC, and Snail1 expression were observed. *H. pylori* directly interacted with the apical surface of apical-out organoids, which increased MUC2 secretion and decreased PGC expression. However, in the floating culture state, relatively weaker PGC expression was observed outside the organoid (**Figure 6D**). Changes in MUC2, PGC, and Snail1 expression were most pronounced in 2D organoids. The single-cell layer structure of 2D organoids, with the apical surface directly exposed, enabled clearer observation of the interaction with *H. pylori* (**Figure 6E**). Therefore, the 2D organoid model is considered a useful platform for overcoming the structural limitations of both 3D and apical-out models (**Figure 6F**). In summary, all organoid models demonstrated increased MUC2 expression in response to *H. pylori* infection, indicating changes associated with intestinal metaplasia in gastric tissue. Decreased PGC expression reflected impaired chief cell function, whereas increased Snail1 expression was associated with EMT activation. Further, *H. pylori* infection significantly increased IL-8 production in all organoid models (**Figure 6G**), indicating an early inflammatory response, most prominent in the apical-out and 2D organoid models. These results indicate that each organoid model plays a complementary role in *H. pylori* infection study. The apical-out model replaces 3D organoid models for studying pathogen interactions, whereas the 2D model, with ALI culture, mimics the actual gastric environment more closely and is useful for showing the infected gastric tissue cross-section.

## 4 Discussion

In this study, we systematically investigated the characteristics and responses of the gastric epithelium to *H. pylori* infection using r-3D, apical-out, and 2D organoid models. Each model exhibits unique advantages and limitations, providing complementary information about the mechanisms of *H. pylori* infection. The adhesion efficiency of *H. pylori* varied based on the structural organization and polarity of the organoids. The apical surface is oriented inward in r-3D organoids, causing relatively low adhesion rates. However, in apical-out and 2D organoids, where the apical surface is directly exposed to *H. pylori*, higher adhesion rates were observed. Particularly, the 2D organoid model, with its fully exposed apical surface, exhibited the most prominent *H. pylori* adhesion. Apical-out organoids maintained a 3D structure while enabling polarity reversal, making them an effective model for investigating pathogen interactions.

*H. pylori* infection-induced inflammatory responses in all organoid models, significantly increasing the expression of IL-8, a proinflammatory cytokine (Eftang et al., 2012; Fazeli et al., 2016). IL-8 upregulation was clearly observed at the early stages of infection in both the apical-out and 2D organoid models. This indicates that these two models are well-suited for investigating the cellular signaling pathways that mediate inflammatory responses in the gastric epithelium. Further, *H. pylori* infection increased MUC2 expression and decreased pepsinogen C expression in all organoid models, reflecting intestinal metaplasia and aberrant differentiation of gastric epithelial cells. MUC2, which is a mucin protein typically expressed in intestinal goblet cells, serves as an intestinal metaplasia marker (Joo and Fang, 2024; Yu et al., 2019). In contrast, pepsinogen C is a marker for gastric chief cells, and its downregulation indicates a loss of gastric differentiation (Kim et al., 2022; Shen et al., 2017; Weis et al., 2014). Furthermore, the upregulation of Snail1, which is a key transcription factor associated with EMT, was observed (Yastrebova et al., 2021; Zhao et al., 2018). Snail1 is involved in E-cadherin downregulation and cellular plasticity promotion, thereby facilitating the transition from an epithelial to a mesenchymal phenotype. These changes were particularly pronounced in the apical-out and 2D organoids, emphasizing the use of these models in studying the pathological alterations caused by *H. pylori* infection.

Both apical-out and 2D organoids effectively visualized apical surface protein redistribution (e.g., ZO-1, a tight junction protein crucial for maintaining epithelial barrier integrity) and the cell–cell junction weakening. *H. pylori* infection decreased ZO-1 expression and TEER in these models, indicating impaired epithelial barrier function. ZO-1 plays a crucial role in regulating tight junctions, and its downregulation is frequently associated with compromised epithelial barrier integrity (Kuo et al., 2021; Tornavaca et al., 2015; Yu et al., 2023). Although 3D organoids preserve structural similarity to *in vivo* tissue, their inward-facing apical surface limits the ability to directly study pathogen–host interactions. In contrast, apical-out organoids combine the benefits of a 3D structure with polarity reversal, enabling robust studies of pathogen interactions. With their simple structure and high reproducibility, 2D organoids are particularly useful for investigating polarity changes and barrier disruption. Further, the inclusion of air-liquid interface (ALI) culture in 2D organoids better mimics the physiological environment of the stomach, making them valuable for examining epithelial barrier function and host–pathogen interactions. In the apical-out organoid model, *H. pylori* infection was maintained for up to 8 hours. While longer incubation times are theoretically possible, extended culture durations may lead to decreased cell viability and a loss of polarity. Therefore, additional optimization of culture conditions would be necessary for long-term infection studies

Noteworthily, the 2D organoid model does not fully replicate the glandular structure of the stomach, and thus its physiological relevance may be limited. This limitation is crucial when considering the complexity of the gastric tissue and the difficulty of fully mimicking its organization in 2D models. The 2D model is valuable for studying physiological features, such as gastric mucus secretion, and the ALI culture improves its ability to mimic the actual stomach environment. Therefore, the 2D organoid model serves as an important tool in *H. pylori* infection research, but its limitations should be acknowledged during result interpretation.

This study demonstrates that both apical-out and 2D organoid models are effective tools for investigating *H. pylori*-host epithelial interactions, inflammatory responses, and pathological changes. The apical-out model provides a 3D platform for studying pathogen interactions through polarity reversal, whereas the 2D model provides a powerful platform for analyzing inflammation and pathology. These models are expected to contribute to a deeper understanding of *H. pylori*-related diseases and the development of future therapeutic strategies.

## Data Availability

The original contributions presented in the study are included in the article/supplementary material, further inquiries can be directed to the corresponding author.
